# Transition to a Moist Greenhouse with CO_2_ and solar forcing

**DOI:** 10.1038/ncomms10627

**Published:** 2016-02-09

**Authors:** Max Popp, Hauke Schmidt, Jochem Marotzke

**Affiliations:** 1 Max Planck Institute for Meteorology, Bundesstrasse 53, Hamburg 20146, Germany; 2 Program in Atmospheric and Oceanic Sciences, Princeton University, 300 Forrestal Road, Sayre Hall, Princeton, New Jersey 08544, USA; 3 Present address: NOAA's Geophysical Fluid Dynamics Laboratory, Princeton, New Jersey, USA

## Abstract

Water-rich planets such as Earth are expected to become eventually uninhabitable, because liquid water turns unstable at the surface as temperatures increase with solar luminosity. Whether a large increase of atmospheric concentrations of greenhouse gases such as CO_2_ could also destroy the habitability of water-rich planets has remained unclear. Here we show with three-dimensional aqua-planet simulations that CO_2_-induced forcing as readily destabilizes the climate as does solar forcing. The climate instability is caused by a positive cloud feedback and leads to a new steady state with global-mean sea-surface temperatures above 330 K. The upper atmosphere is considerably moister in this warm state than in the reference climate, implying that the planet would be subject to substantial loss of water to space. For some elevated CO_2_ or solar forcings, we find both cold and warm equilibrium states, implying that the climate transition cannot be reversed by removing the additional forcing.

## Abstract (web summary)

Greenhouse-gas forcing has previously been thought to be rather ineffective at destroying the habitability of Earth-like planets. Here, the authors show that CO_2_ is as effective as solar forcing at causing a climate transition to a Moist-Greenhouse regime and thus poses an equal threat to a planet's habitability.

## Graphical abstract

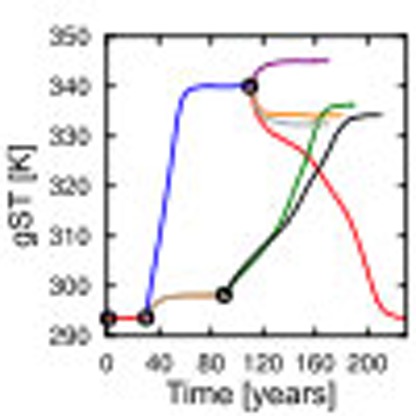

Water-rich planets such as Earth lose water by photo-dissociation of water vapour in the upper atmosphere and the subsequent escape of hydrogen. On present-day Earth, the loss occurs very slowly, because the mixing ratio of water vapour in the upper atmosphere is very low. But significant loss of water could occur over geological timescales if the surface temperature were around 70 K warmer than it is today^2^,^3^,^4^. For these high surface temperatures, the tropopause is expected to climb to high altitudes. As a consequence, the cold trapping of water vapour at the tropopause becomes ineffective, because the mixing ratio of water vapour increases with the rising tropopause. Steady states in which the mixing ratio in the upper atmosphere is sufficiently high for a water-rich planet to lose most of its water inventory in its lifetime are known as Moist-Greenhouse states^5^. A planet in this state would eventually become uninhabitable as all water is lost to space. For an Earth-like planet around a Sun-like star, a Moist Greenhouse would be attained if the mixing ratio in the upper atmosphere exceeds ∼0.1 % (ref. 1). For comparison, the mixing ratio in Earth's stratosphere is presently around two orders of magnitude smaller.

Moist-Greenhouse states were found and described in several studies with one-dimensional models^2^,^3^,^4^,^6^,^7^ and have recently been found for terrestrial planets with three-dimensional models in different setups^8^,^9^,^10^. However, not all three-dimensional studies found stable Moist-Greenhouse states^11^,^12^,^13^. Instead the climate of these models would destabilize into a Runaway Greenhouse, a self-reinforcing water-vapour feedback-loop, before the Moist Greenhouse is attained. A few studies applied large forcing but the employed models became numerically unstable before the Moist-Greenhouse regime was attained^14^,^15^,^16^. Therefore, it remains unclear whether planets would attain a Moist-Greenhouse state before a Runaway Greenhouse occurs, especially for planets on an Earth-like orbit, where the only two previous studies with state-of-the-art general circulation models (GCM) gave contradicting results^13^,^10^. Moreover, all three-dimensional studies investigating Moist-Greenhouse states only applied solar forcing without considering greenhouse-gas forcing. Several studies have applied strong greenhouse-gas forcing, but either did not run their simulations to sufficiently high temperatures^17^,^18^,^19^,^20^ or did not investigate the emergence of a Moist Greenhouse^21^. Greenhouse-gas forcing has long been assumed to be ineffective at causing Moist-Greenhouse states, because the greenhouse effect of any additional greenhouse gas would eventually be rendered ineffective by the increasing greenhouse effect of water vapour with increasing temperatures. Furthermore, large greenhouse-gas forcing would lead to a cooling of the upper atmosphere, which would push the Moist-Greenhouse limit to much higher surface temperatures^6^. However, if clouds are considered, these arguments may not apply, because clouds themselves can contribute to the climate becoming unstable^22^.

Here we compare for the first time with a state-of-the-art GCM, namely ECHAM6 (ref. 22), how effective solar and CO_2_ forcing are at causing a transition to a Moist Greenhouse. We couple the atmosphere to a slab ocean and choose an aqua-planet setup (fully water-covered planet) in perpetual equinox. This idealized framework is better suited than a present-day Earth setting to understand the involved dynamics while preserving the major feedback mechanisms of the Earth^24^. It also avoids conceptual problems with the representation of land-surface processes at high temperatures. We turn off sea ice in order to investigate the possibility of solely cloud-induced multiple steady states that were recently found in a one-dimensional study^22^. We modify the model such that it can deal with surface temperatures of up to 350 K (see Methods).

Thus we show that cloud-radiative effects (CRE) destabilize a present-day Earth climate as readily with CO_2_ as with solar forcing. The changes in CRE are a consequence of the weakening of the large-scale circulation with increasing global-mean surface temperature (gST). However, the resulting climate transition does not lead to a Runaway Greenhouse, but instead a new regime of warm steady state with gST above 330 K is attained. This warm regime differs substantially in its dynamics from a present-day Earth-like climate and, most importantly, the upper atmosphere exceeds the Moist-Greenhouse limit in this regime. Hence a planet in such a state would lose water at a fast rate to space. Furthermore, there is hysteresis in the warm regime and removing the imposed forcing does therefore not necessarily cause a transition back to an Earth-like climate.

## Results

### Simulations with increased TSI

**Figure 1:**
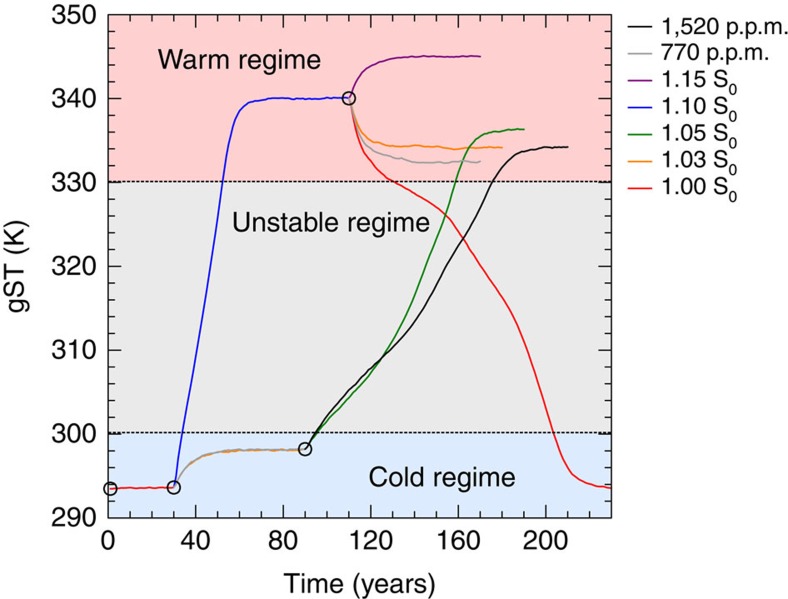
Temporal evolution of gST. The circles denote the four states from which new simulations are started. For both a TSI of 1.00 S_0_ and 1.03 S_0_ as well as for a CO_2_ concentration of 770 p.p.m., two simulations with different initial conditions are performed. The lines are interpolated from annual means.

**Figure 2:**
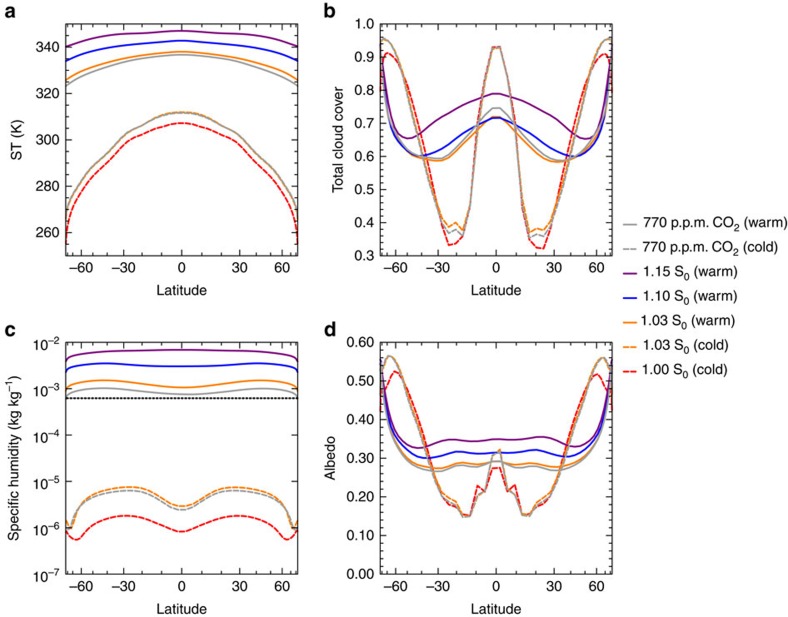
Zonal means in steady state. (**a**) Surface temperature (ST); (**b**) the total cloud cover; (**c**) the specific humidity at the top level; and (**d**) the effective albedo. The effective albedo is defined as the ratio of the zonal and temporal means of the reflected solar radiation divided by the zonal and temporal means of the incoming solar radiation. The black horizontal line in **c** indicates the Moist-Greenhouse limit^2^. The temporal mean is taken over a period of 30 years. The horizontal axes are scaled with the sine of the latitude.

To assess the dependence of the climate state on total solar irradiance (TSI) for fixed CO_2_ levels (at 354 p.p.m. volume mixing ratio), we apply a total of five different TSI-values that range from the present-day value on Earth (S_0_) to 1.15 times that value. We find two regimes of steady states that are separated by a range of gST for which stable steady states are not found (Fig. 1). The regime of steady states with gST of up to ∼298 K exhibits similar features as present-day Earth climate, such as a large pole-to-equator surface-temperature contrast (Fig. 2a) and a similar meridional distribution of cloud cover (Fig. 2b). Hence this regime of steady state can be considered to be Earth-like. In contrast, the warm regime of steady states with gST above 334 K is characterized by a considerably smaller pole-to-equator surface-temperature difference and a substantially different meridional distribution of cloud cover (Fig. 2a,b). This illustrates that the dynamics in the warm regime are quite different from the present-day Earth regime. Most importantly, the mixing ratio of water vapour at the uppermost level at 0.01 hPa is considerably higher in the warm regime and exceeds the Moist-Greenhouse limit (Fig. 2c). Therefore, a planet in such a state would be losing water to space at a fast rate. The minimum TSI required to cause a climate transition from the Earth-like to the warm regime lies between 1.03 S_0_ and 1.05 S_0_, whereas the maximum TSI to cause a climate transition from the warm back to the Earth-like regime lies between 1.00 S_0_ and 1.03 S_0_ (Fig. 1). Consequently, there are two different stable steady states for a TSI of 1.03 S_0_. Since sea ice is turned off in our model, this double steady state is entirely a consequence of atmospheric processes. In the warm regime, the cloud albedo increases at all latitudes with TSI, thus providing an efficient way to stabilize the climate against increased radiative forcing (Fig. 2d).

### Energetics of the climate transition

**Figure 3:**
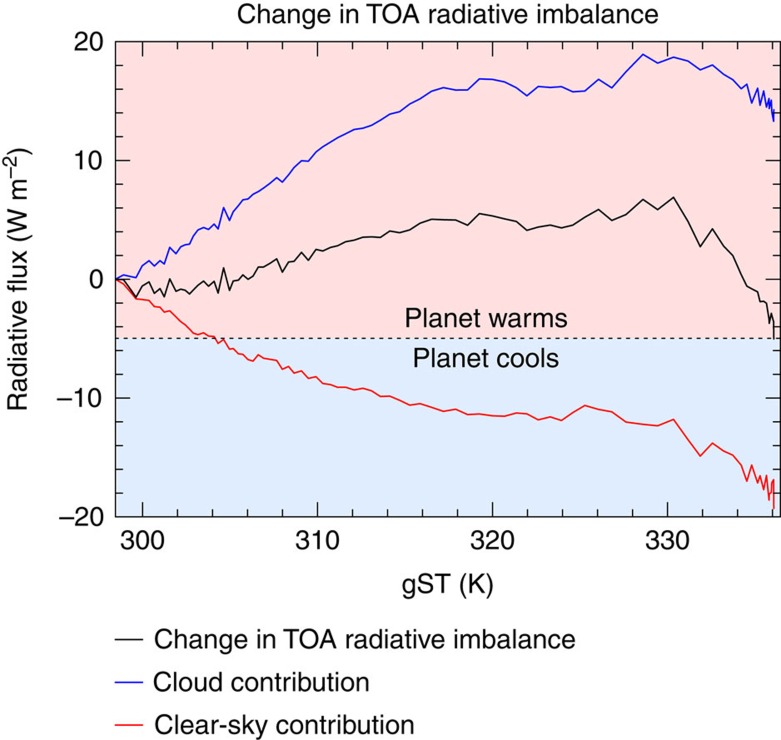
Change of TOA radiative flux as a function of gST. The black line shows the change in global and annual mean of the total (shortwave plus longwave) net (downward minus upward component of the) TOA radiative flux, the blue line shows the cloud-radiative contribution and the red line the clear-sky contribution from the transient simulation with a TSI of 1.05 S_0_. Note that a positive slope indicates an unstable state, because any warming/cooling would lead to an increase/decrease in energy uptake by the aqua-planet and thus to an additional warming/cooling. The cloud-radiative and clear-sky contributions sum up to the net TOA radiative flux. The changes are calculated by subtracting the global and annual means over the first year of the respective quantities. The horizontal line corresponds to the negative value of the initial total net TOA radiative flux. Hence, for a steady state to be attained, the total net TOA radiative flux must touch or intersect the horizontal line.

To understand the processes governing the climate transition from the cold to the warm regime, we focus now on the transient simulation with a TSI of 1.05 S_0_. The climate instability is evidenced by an increase in the total (shortwave plus longwave) net (downward minus upward component of the) global-mean top-of-the-atmosphere (TOA) radiative flux with increasing gST for gST between 300 and 330 K (Fig. 3). Note that a positive TOA radiative flux is net downward and a negative radiative flux is net upward. Therefore, the aqua-planet takes up energy for a positive TOA radiative flux and loses energy for a negative flux. The instability is caused by the cloud-radiative contribution to the total radiative flux that increases with increasing gST for gSTs below 330 K. The clear-sky contribution to the total radiative flux is decreasing with increasing gST and does thus not contribute to the climate instability. This decrease in clear-sky contribution is caused by an increase in clear-sky contribution to the outgoing longwave radiation, which is upward and thus decreases the net downward flux. At gST above 330 K, the cloud-radiative contribution decreases again with increasing gST. This allows together with the clear-sky contribution to attain a new steady state. Thus, clouds destabilize the climate at lower gST and then stabilize again at higher gST. This change in sign of the cloud feedback is also responsible for the existence of the bistability.

### Dynamics of the climate transition

**Figure 4:**
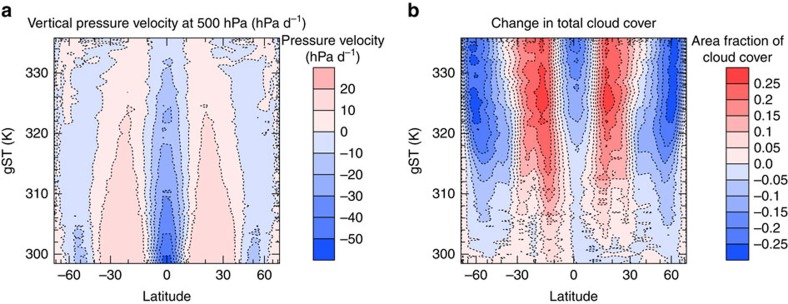
Large-scale circulation and cloud cover during the climate transition. (**a**) Zonal and annual mean of the vertical pressure velocity as a function of latitude and gST for the transient period of the simulation with a TSI of 1.05 S_0_. (**b**) Same as in **a** but for the change in zonal and annual mean of cloud cover from the first year of simulation.

**Figure 5:**
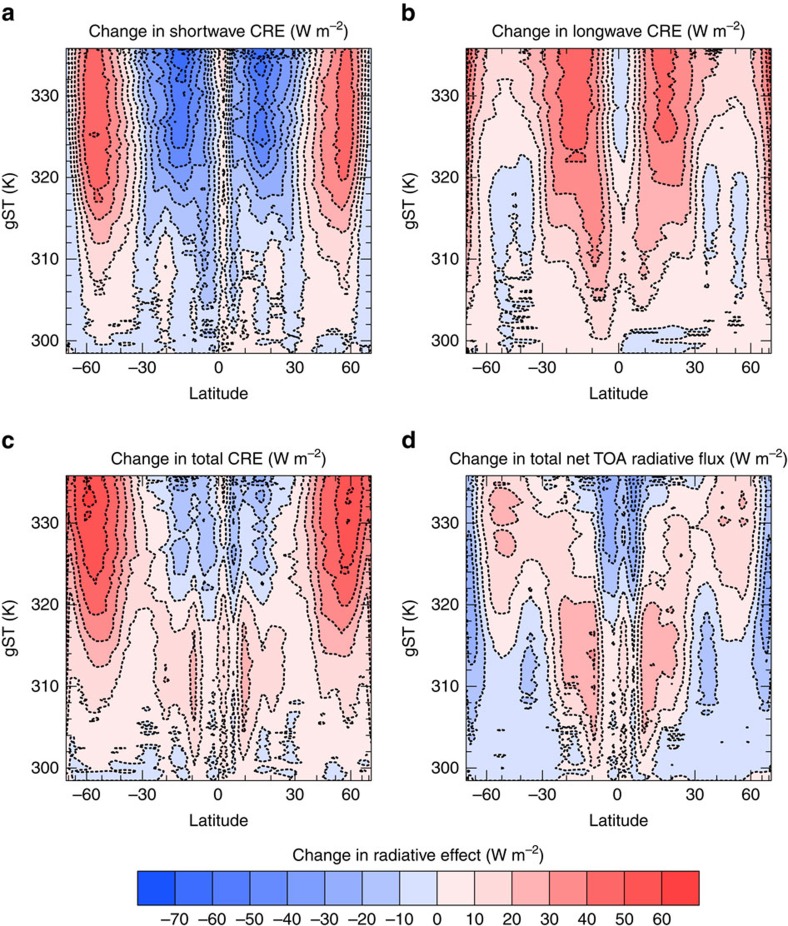
Zonal means of changes in radiative effects during the climate transition. (**a**) Change of zonal and annual mean of the shortwave CRE; (**b**) change of the longwave CRE; (**c**) change of the total CRE; and (**d**) the change of the total net TOA radiative flux. All panels use the same colour bar. The horizontal axes are scaled with the sine of the latitude.

**Figure 6:**
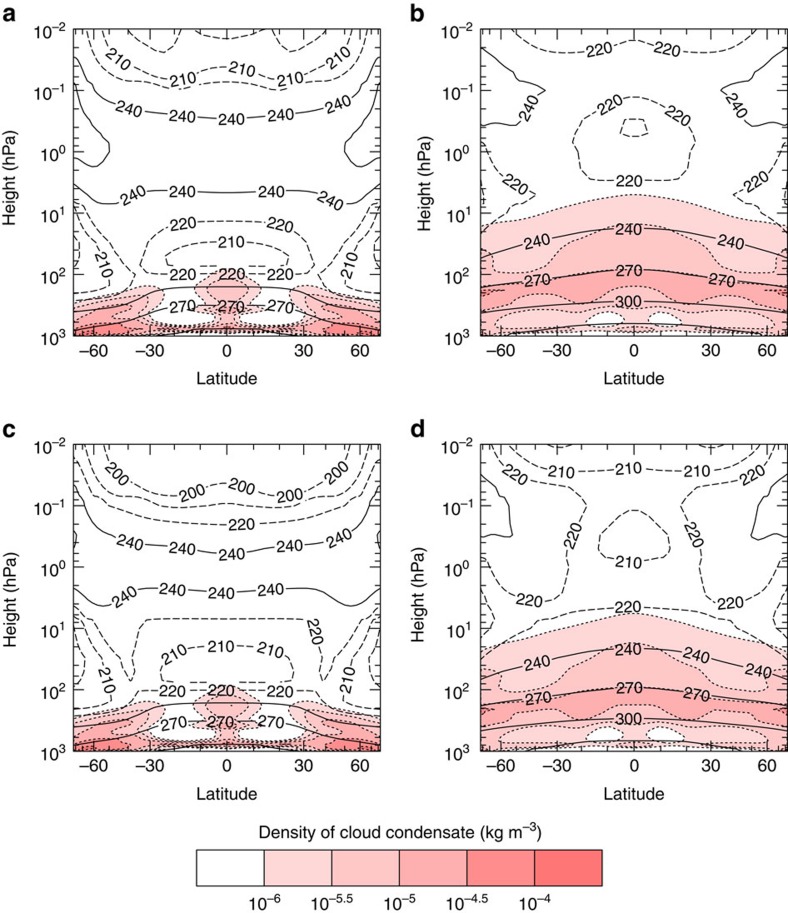
Zonal means of cloud condensate in steady state. (**a**) Temporal mean over the last 30 years of simulation of the cloud condensate for a TSI of 1.03 S_0_ in the cold regime and (**b**) same quantity for the same TSI but in the warm regime. (**c**) Same quantity obtained with a TSI of 1.00 S_0_ but with atmospheric CO_2_ concentrations of 770 p.p.m. in the cold regime (**d**) with the same CO_2_ concentration and TSI in the warm regime. The contours denote temperatures in Kelvin, with the solid lines denoting the contours for 240, 270, 300 and 330 K and the dashed lines for the contours of 200, 210 and 220 K. The vertical axes are the height in terms of pressure of dry air and the horizontal axes are the latitudes scaled with their sines. The pressure of dry air is defined to be the pressure the atmosphere would have at a given level if no water vapour was present. Since the total mass of dry air does not change between simulations, the mean of the dry surface pressure is constant across simulations.

The changes in cloud-radiative contribution to the total net TOA radiative flux (henceforth simply referred to as CRE) are caused by the weakening of the large-scale circulation (Fig. 4a) and the increase of water vapour in the atmosphere with increasing gST. The weakening of the circulation causes tropical convection to spread more evenly around the tropics, with less convection occurring around the equator and more convection occurring in the subsidence region. As a consequence, deep convective clouds with low cloud-top temperatures become more frequent in the subsidence region of the Hadley circulation (Fig. 4b). This in turn leads to a very strong increase in longwave CRE in this region, which dominates the increase in shortwave CRE (Fig. 5a,b). However, as the gST increases further and the specific humidity in the atmosphere increases, the clouds become thicker and thus more reflective, whereas the longwave CRE does not increase as fast anymore leading to a decrease in total CRE in the tropics for gST above 320 K. This decrease in tropical CRE with increasing gST contributes to the stabilization of the climate at gST above 330 K (Fig. 5c). In general, the changes in total CRE dominate the changes in clear-sky radiative effect in the tropics (Fig. 5c,d). In the extra-tropics, the weakening of the large-scale circulation leads to a steady decrease in cloud cover everywhere except at very high latitude (Fig. 4a,b). Therefore, the shortwave CRE increases in the extra-tropics (Fig. 5a). Since the tropopause deepens with increasing surface temperatures (not shown), the difference between the temperature at the surface and at the cloud tops increases as well and leads also to an increase in longwave CRE despite the decrease in cloud cover (Fig. 5b). Whereas the changes in the clear-sky radiative effect dominate the changes in CRE in most of the extra-tropics for gST up to 315 K, the changes in CRE increasingly dominate the extra-tropical response at gST above. This supports the idea that at high gST changes in CRE are more important than changes in clear-sky radiative effect and dominate the climate response. In general, the weak large-scale circulation in the warm regime leads to a much more uniform meridional distribution of cloud condensate at all levels than in the cold regime (Fig. 6). Note that there is a decrease in global-mean convective precipitation but a slight increase in total precipitation from the cold to the warm regime (not shown). This may indicate that in the warm regime convection is overall less frequent but more intense, such that a significant fraction of condensate is not converted to convective precipitation but detrained to form large-scale precipitation. This trend continues as surface temperatures increase further in the warm regime.

### Simulations with increased CO_2_ concentrations

We start our comparison of CO_2_-induced to solar forcing by increasing CO_2_ concentrations to 770 p.p.m., while keeping the TSI fixed to 1.00 S_0_. This corresponds to an equivalent adjusted forcing as is caused by an increase of TSI from 1.00 S_0_ to 1.03 S_0_. The adjusted forcing is defined to be the temporal and global mean of the energy uptake over the first year of simulation. The results suggest that the increase of the CO_2_ concentrations leads to an equivalent warming and a similar meridional distribution of surface temperatures and clouds as the increase in TSI does (Fig. 2a,d). Since the aqua-planet warms by 4.57 K for an increase in CO_2_ concentrations from 354 to 770 p.p.m., the climate sensitivity of the aqua-planet for a doubling of CO_2_ concentrations is 4.08 K, if a log_2_ scaling is assumed. Starting from the final state of the simulation with a TSI of 1.03 S_0_, we then increase the CO_2_ concentrations to 1,520 p.p.m. and set the TSI back to 1.00 S_0_ (Fig. 1). The combined effect leads to an adjusted forcing that is equivalent to increasing the TSI to 1.05 S_0_. In this case the aqua-planet undergoes a climate transition into the warm regime (Fig. 1). Thus, the aqua-planet can as readily be forced to transition from the Earth-like to the warm regime by increasing CO_2_ concentrations as by increasing the TSI. When starting in the warm regime, a reduction of CO_2_ concentrations to 770 p.p.m. does not cause the planet to fall back into the Earth-like regime, but the aqua-planet remains in the warm regime. Therefore, the aqua-planet also exhibits a bistability of the climate for a TSI of 1.00 S_0_ and a CO_2_ concentration of 770 p.p.m.

Overall, the results suggest that the aqua-planet behaves similarly for solar forcing and CO_2_-induced forcing (Figs 1, 2 and 6). The most notable difference is that the steady-state gST in the warm regime is ∼2 K lower for CO_2_-induced forcing. The likely reason for this is that the thermal absorption by water vapour overlaps with the thermal absorption by CO_2_ in the warm moist atmosphere, which renders the greenhouse effect of CO_2_ less effective. However, since the climate instability in our simulations is caused by CRE at a gST at which the atmosphere is not yet sufficiently opaque to cancel the greenhouse effect of CO_2_, CO_2_-induced forcing can as easily cause a climate transition to the Moist Greenhouse as solar forcing does.

### Sensitivity experiments

Two of the assumptions made in this study could potentially have a large influence on the results and may contribute to the differences in the results between our study and previous ones^18^,^13^,^10^,^21^. These assumptions concern the treatment of ozone and oceanic heat transport of the model. For these two cases, we show with sensitivity experiments that the qualitative nature of the results is not changed by the assumptions (Supplementary Figs 1–3). The neglect of sea ice should have no influence on the qualitative results, because there is no sea ice in the warm regime and because the sea-ice albedo feedback is positive and would thus favour a climate instability in the cold regime. However, the absence of sea ice may explain why our control simulation is warmer than present-day Earth.

## Discussion

A recent study using the same model but in a different version found that the Earth's climate remains stable for CO_2_ concentrations of at least 4,480 p.p.m. (ref. 17), whereas our study suggests that such concentrations would lead to a climate transition. Studies of Earth with other GCMs also found the climate to remain stable for higher CO_2_ concentrations than we do^17^,^19^. However, the initial climate of our aqua-planet is ∼6 K warmer than the one of present-day Earth. Such a warming would be attained by a quadrupling of CO_2_ in the different version of our model used in ref. 17. By a simple estimate, this other study would thus have explored CO_2_ concentrations of up to a fourth of 4,480 p.p.m.; hence, 1,120 p.p.m., if the simulations were started from a climate similar to ours. Therefore, if we account for the difference in the initial climates, the results of the two studies are not in contradiction. Indeed, the climate of the model version used in ref. 17 was recently shown to become unstable when the CO_2_ concentrations were increased from 4,480 to 8,960 p.p.m. (eventually leading to numerical failure of their model)^20^. Nonetheless, the forcing required to cause a climate transition would certainly be higher on present-day Earth than on our aqua-planet, even with our version of the model. Several other studies of Earth have found lower climate sensitivities to relatively large CO_2_ forcing than we do which supports this notion^17^,^19^,^21^.

**Figure 7:**
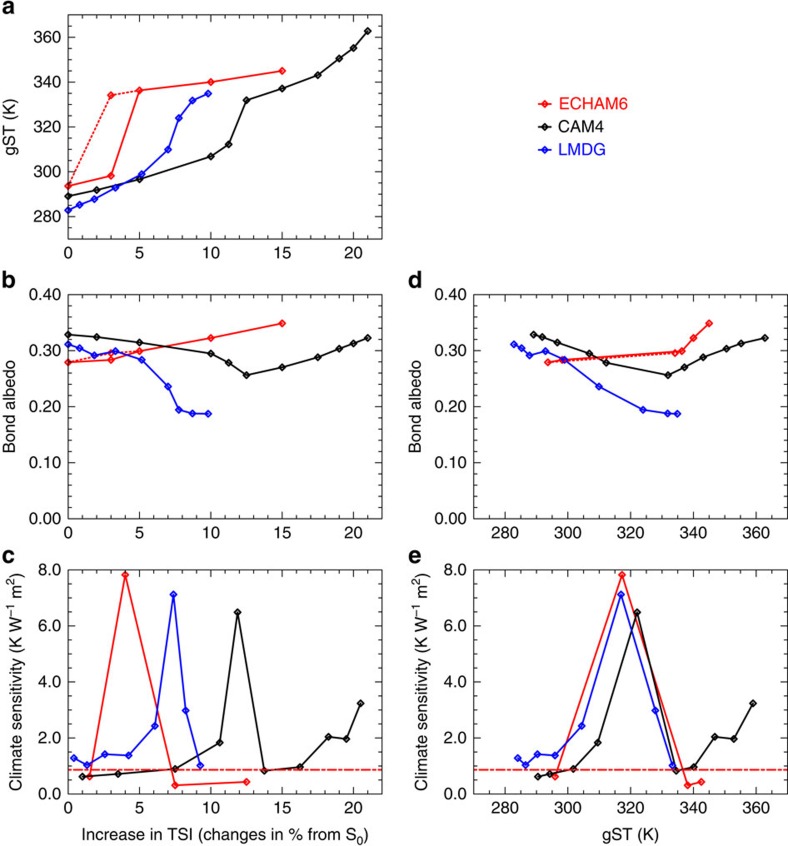
Model inter-comparison. (**a**) gST; (**b**) the Bond albedo; and (**c**) the climate sensitivity as a function of the increase in TSI in per cents of S_0_. The red solid lines and marks denote the results obtained with ECHAM6 when increasing TSI, whereas the dashed lines and mark denote the results obtained with ECHAM6 but when decreasing TSI. The black lines and marks denote the results obtained with CAM4 in ref. 9 and the blue lines and marks denote the results obtained with LMDG in ref. 12. (**d**) Bond albedo and (**e**) the climate sensitivity as a function of the gST. The red dashed-dotted line denotes the climate-sensitivity parameter for doubling CO_2_ and is calculated from the simulation in which CO_2_ concentrations are increased from 354 to 770 p.p.m. using log_2_ scaling. Note that the Bond albedo is equal to the global-mean of the effective albedo.

**Figure 8:**
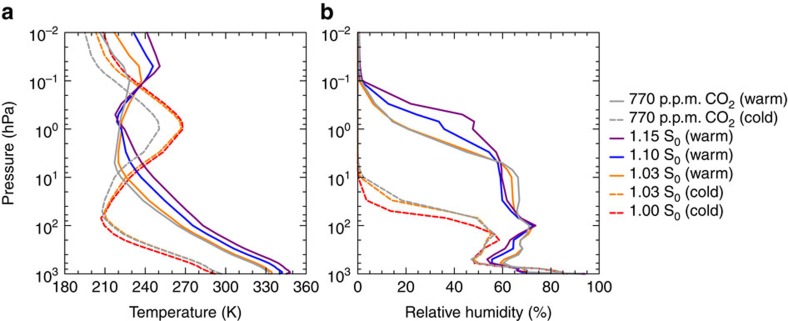
Atmospheric profiles of temperature and relative humidity. (**a**) Global-mean vertical profiles of gST and (**b**) of relative humidity for the different steady states. The vertical axes are the height in terms of pressure of dry air.

Two studies recently investigated climates at gST above 330 K with state-of-the-art GCMs for Earth-like planets^13^,^10^. Wolf and Toon^10^ used a modified version of the Community Atmosphere Model version 4 (CAM4) and Leconte *et al*.^13^ a modified version of the Laboratoire de Météorologie Dynamique Generic (LMDG) climate model to investigate the climate response to strong solar forcing and both studies also found a region of increased climate sensitivity^13^,^10^. Therefore, a region of gST with increased climate sensitivity surrounded by regions of lower climate sensitivity appears to be a robust result, despite some differences in the magnitude of the region (Fig. 7c,e). We calculated the climate sensitivity here following^10^, which yields a small climate sensitivity in the cold and in the warm regimes because the method uses the instantaneous forcing. If we use the adjusted forcing, the climate sensitivity is considerably larger, because the fast atmospheric adjustments reduce the initial TOA radiative imbalance quickly. Our model encounters the region of high climate sensitivity for smaller values of TSI (Fig. 7a), because our control climate is the warmest and because the climate instability is encountered at ∼300 K, whereas the region of increased climate sensitivity starts at ∼310–315 K in the two other studies. Our steady-state albedo is a monotonically increasing function of both TSI and gST, in contrast to the two other studies where the albedo decreases with both increasing TSI and gST until either the Moist-Greenhouse state is attained or a Runaway Greenhouse occurs (Fig. 7b,d). However, in our transition from the cold to the warm regime the albedo decreases as well with increasing gST between 300 and 320 K. Only one of the two studies found a stabilizing cloud feedback similar to ours and a moist stratosphere at high gST (ref. 9), whereas the other one found that the cloud feedback is rather destabilizing and that the stratosphere remains dry^13^. In general, our results of the simulations with increased solar forcing are qualitatively similar to the ones found in ref. 9 in that we find two regimes of steady states, in that the warm regimes have a similar temperature structure (Fig. 8a), in that the cloud albedo increases with gST and most importantly in the existence of a stable Moist-Greenhouse regime. Similarly to ref. 9, the troposphere in the warm regime is characterized in our model by a particular radiative-convective equilibrium with a temperature inversion close to the surface, a somewhat drier region above and a more humid region up to the tropopause. A similar structure has also been found and discussed in two one-dimensional studies^6^,^22^. Wolf and Toon^10^ argues that the change to the aforementioned radiative-convective equilibrium is crucial for the emergence of stable Moist-Greenhouse states, but our results suggest that the weakening of the large-scale circulation is equally important by allowing the radiative-convective regime to spread over the entire tropics. This spread of the convective region over a large fraction of the planet, namely the tropics, also explains some of the similarities between the three- and the one-dimensional models in the warm regime. Compared with ref. 9, the dry region is more humid in our warm regime and in general more humid than in our cold regime (Fig. 8b, Supplementary Fig. 4). Given the different dynamical cores, radiative transfer schemes, convection schemes and cloud schemes, it is, however, remarkable how many similarities the three models share.

In the two aforementioned studies of large solar forcing, ozone was entirely removed from the whole atmosphere in all simulations^13^,^10^, whereas we do include ozone in our calculations. Since ozone has a strong warming influence in the middle atmosphere due to its strong absorption of solar radiation, the inclusion of ozone in our model may explain why our atmosphere is moister in the upper levels than theirs for similar values of gSST. To test this hypothesis, we perform a simulation with a TSI of 1.10 S_0_ without atmospheric ozone. Both the upper atmospheric temperatures and specific humidity are lower but still above the Moist-Greenhouse limit in the experiments where ozone is removed (Supplementary Fig. 1). Thus, the specific humidity is still considerably higher than in our cold regime and especially than in the simulations of Wolf and Toon^10^ and Leconte *et al*.^13^ at similar gSST. In general, removing ozone does not appear to alter the results substantially.

One study investigated strong CO_2_ forcing and gST above 330 K with the Fast Atmosphere-Ocean Model (FAOM) developed at the Goddard Institute for Space Studies and did not find any region of strongly increased climate sensitivity^21^. The climate sensitivity increases somewhat but not nearly as much as with our or with the other two state-of-the-art models used for solar forcing. As a consequence, considerably higher values of CO_2_ are required in that study compared with ours to attain a gST of 330 K. Furthermore, the control climate is colder in that study, and it takes around one doubling of CO_2_ to attain the gST of our control simulation. The humidity at the top level of FAOM remains roughly one order of magnitude below the Moist-Greenhouse limit in the warmest steady states found in ref. 20. This may be partly due to the CO_2_ cooling of the upper atmosphere, but could also be a consequence of not running the model to sufficiently high temperatures. We perform a sensitivity experiment, where we increase CO_2_ concentrations to 9,000 p.p.m. in order to assess whether increased CO_2_ concentrations could cause a substantial drying of our upper atmosphere (not shown). The upper atmosphere-specific humidity stays; however, well above the Moist-Greenhouse limit also in that case. Some of the differences between FAOM and ECHAM6 may simply be caused by the use of different setups and parameterizations. But FAOM is a simplified model designed for fast computation and uses simplified cloud physics, which may be the cause for the absence of a region of strongly increased climate sensitivity. So, whereas our version of ECHAM6 is rather on the low side of CO_2_ concentrations required to cause the gST to rise above 330 K, FAOM likely is on the high side of the concentrations.

To conclude, we have demonstrated with a state-of-the-art climate model that a water-rich planet might lose its habitability as readily by CO_2_ forcing as by increased solar forcing through a transition to a Moist Greenhouse and the implied long-term loss of hydrogen. We confirm previous results that a region of increased climate sensitivity exists and show that the climate is unstable in our model in that region due to positive cloud feedbacks caused by a weakening of the large-scale circulation. We also demonstrate that there is hysteresis and that once a transition to the Moist-Greenhouse regime has occurred, the process may not simply be reversed by removing the additional forcing.

## Methods

### General setup

We employ a modified version of the GCM ECHAM6 (ref. 22) in an aqua-planet setting in which the whole surface is covered by a 50-m-deep mixed-layer ocean. We run the model with a spectral truncation of T31, which corresponds to a Gaussian grid with a grid-point spacing of 3.75°. The atmosphere is resolved vertically by 47 layers up to a pressure (of dry air) of 0.01 hPa. The oceanic heat transport is prescribed by a sinusoidal function of latitude. There is no representation of sea ice in our model and as a consequence water may be colder than the freezing temperature. The orbit of the aqua-planet is perfectly spherical with a radius of 1 AU. The obliquity of the aqua-planet is 0°. For simplicity, a year is set to be 360 days. The rotation velocity of the aqua-planet corresponds to present-day Earth.

### Parameterized oceanic heat transport

To account for the meridional oceanic heat transport, we introduce an additional energy flux from the mixed-layer ocean to the atmosphere, which mimics the divergence of a prescribed meridional oceanic heat flux. This energy flux is commonly called q-flux. Imposing a q-flux is necessary to prevent the atmosphere from having to take over all the meridional energy transport, which would result in an amplified atmospheric large-scale circulation. However, since the oceanic circulation depends also on atmospheric properties, the imposed q-flux may be inaccurate in a warming climate. The weak atmospheric large-scale circulation suggests that the oceanic heat transport would be weaker in the warm regime and that thus the absolute values of the q-flux are too large. To assess whether the imposed q-flux affects the results in the warm regime, a number of simulations without q-flux are performed. Despite small differences in the large-scale circulation (Supplementary Fig. 2), the warm steady states exist and are stable even without q-flux (Supplementary Fig. 3).

### Treatment of ozone

If the tropopause climbs, regions with high ozone concentrations may come to lie in the troposphere of the model, because ozone concentrations are prescribed to climatological values. High ozone concentrations could, however, not occur in the presence of tropospheric water vapour concentrations. Therefore, we limit the tropospheric ozone concentrations to a volume mixing ratio of 1.5 × 10^−7^. As a consequence, ozone is taken out of the atmosphere, if the tropopause rises to levels where the climatological values would exceed this limit. This process is reversible if the tropopause descends again.

### Modifications to the grid-point physics

Our version of the model incorporates several changes to the grid-point physics, such that we obtain a more accurate representation of several physical processes in warm climates^22^. The grid-point physics include representation of surface exchange, turbulence and vertical diffusion^25^,^26^, gravity-wave drag^27^,^28^, radiative transfer and radiative heating^29^,^30^, convection^31^,^32^, cloud cover^33^ and cloud microphysics^34^. In summary, these changes are the inclusion of the mass of water vapour when calculating the total pressure and the omission of all approximations where small specific humidities are assumed (as for example in the calculation of density). The pressure effects of water vapour are not considered for the horizontal transport. So, the model is in sorts a hybrid model, with water vapour adding to the total pressure for local effects but not so for the large-scale transport. A detailed description of the modified model thermodynamics can be found in the appendix of ref. 34. We will give here a short overview of the modified radiative transfer scheme as well as of the convection, cloud-cover and cloud-microphysical schemes.

### Radiative transfer

The radiative transfer scheme has recently been described and evaluated extensively in ref. 21, but as a courtesy to the reader we will repeat some of the major features here. It is based on the Rapid Radiative Transfer Model^29^,^30^, but includes some small modifications. It uses the correlated-k method to solve the radiative transfer equations in the two-stream approximation. The k-coefficients are calculated from the HITRAN (1996 and 2000) database using a line-by-line radiative transfer model^29^,^30^. The water vapour continuum is based on CKD_v2.4. The shortwave radiation spectrum is divided into 14 bands, and the longwave radiation spectrum is divided into 16 bands. Since the lookup-tables of the molecular absorption coefficients are designed for a limited range of temperatures only, an exponential extrapolation for temperatures up to 400 K for the longwave radiation scheme is performed. The same extrapolation scheme is also applied to the lookup-tables for the absorption coefficients of the water vapour self-broadened continuum in the shortwave radiation scheme, but the original linear extrapolation scheme is kept for all the other absorption coefficients. The lookup-tables for the bandwise spectrally integrated Planck function and the derivative thereof with respect to temperature have been extended to 400 K. Furthermore the water vapour self-broadened continuum is introduced in the upper atmosphere radiation calculations, to account for the increase in water vapour with increasing gST. The effect of pressure broadening by water vapour on the molecular absorption coefficients is neglected, as is scattering by water vapour. The thus modified radiation scheme is not as accurate as line-by-line radiative transfer models or models using recalculated k-coefficients for the higher temperatures, but still sufficiently accurate for the task at hand as has recently been demonstrated^22^.

### Convective scheme

ECHAM6 uses a mass-flux scheme for cumulus convection^31^, with modifications for penetrative convection to the original scheme^32^. The contribution of cumulus convection to the large-scale budgets of heat, moisture and momentum is represented by an ensemble of clouds consisting of updrafts and downdrafts in a steady state. Depending on moisture convergence at the surface and depth of the convection cell, the model will either run in penetrative, mid-level, or shallow convection mode. The scheme allows for the formation of precipitation, but not for radiatively active convective clouds. Instead, detrained water is passed to the cloud-microphysical scheme which creates or destroys cloud condensate in a further step.

### Cloud-cover scheme

ECHAM6 uses the Sundqvist scheme for fractional cloud cover^33^. This cloud scheme has been tuned for the use with present-day Earth's climate^34^,^35^. However, the scheme is well suited for simulations of cloud cover in warm climates as it diagnoses cloud cover directly from relative humidity, which should be crucial to cloud formation irrespective of the temperature.

### Cloud-microphysical scheme

The cloud-microphysical scheme is described in detail in ref. 33. The scheme consists of prognostic equations for the vapour, liquid, and ice phases. There are explicit microphysics for warm-phase, mixed-phase and ice clouds. The cloud-condensation-nuclei concentration follows a prescribed vertical profile which is typical for the present-day Earth's maritime conditions. Since we have no estimate of the aerosol load in a hypothetical warm climate, we assume that this profile is also a reasonable choice for warmer climates. The microphysics do not require changes for the use of the scheme in warm climates, since cloud formation in a warm-phase cloud, in which potential changes may occur, is not directly temperature-dependent (at least not in the range of temperatures we consider).

### Cloud-radiative interactions

Clouds are represented in the radiative transfer calculations, assuming the so-called maximum-random-overlap assumption. Under this assumption cloud layers are assumed to be maximally overlapping if they are adjacent to one another, and randomly overlapping if they are separated by a clear layer. The absorptivity of clouds depends on their combined optical depths, the gas in which they are embedded, and the interstitial aerosol. The microphysics to determine the optical properties of the cloud particles involve the liquid water and ice paths, cloud-drop radii, as well as liquid water and ice content. Cloud scattering is represented as a single-scattering albedo by assuming Mie scattering from cloud droplets in the shortwave calculations, but is neglected in the longwave calculations. Clouds are not considered in the radiative transfer routines if the specific mass of the cloud condensate does not exceed 10^−7^ kg per kg of air.

### Special settings for high-temperature simulations

To run the model at high temperatures, a few special settings are necessary. The time step is reduced from 2,400 to 600 s, and the radiation time step is reduced from 7,200 to 2,400 s, except for the simulation with a TSI of 1.15 S_0_, where the time step is reduced to 360 and the radiation time step to 1,440 s. Nonetheless, we sometimes encounter problems with resolved waves propagating to the top levels of the model, where their amplitude grows and where they may be reflected. To avoid frequent model failure due to these effects, we introduce Rayleigh friction to the vorticity and the divergence as well as increased horizontal diffusion in the top six layers (above ∼0.75 hPa). The time constant of the Rayleigh friction is (10800s) at the sixth layer and is increased by a factor of 3.2 per layer towards the top and is hence increased by a factor of ∼1,000 in the top layer. The horizontal diffusion is increased by a factor of 3.2 per layer. The values of the time constant of the Rayleigh friction and the magnitude of horizontal diffusion are determined by trial and error. Since we investigate a large range of climates, it is difficult to find suitable values for these time constants, and despite these modifications the model fails occasionally. In these cases, however, the runs can be continued by slightly changing their trajectory, which is achieved by reducing the factor of multiplication per level for the Rayleigh friction from 3.2 to 3.199 for 30 days.

## Additional information

**How to cite this article:** Popp, M. *et al*. Transition to a Moist Greenhouse with CO_2_ and solar forcing. *Nat. Commun.* 7:10627 doi: 10.1038/ncomms10627 (2016).

## Supplementary Material

Supplementary Information Supplementary Figures 1-4
