# Supplementary material for: Transition to a Moist Greenhouse with CO2 and solar forcing
Source: Nat Commun. 2016 Feb 9;7:10627. doi: 10.1038/ncomms10627 (PMC4748134; doi:10.1038/ncomms10627)
Supplement: Supplementary Information — Supplementary Figures 1-4 [file ncomms10627-s1.pdf]

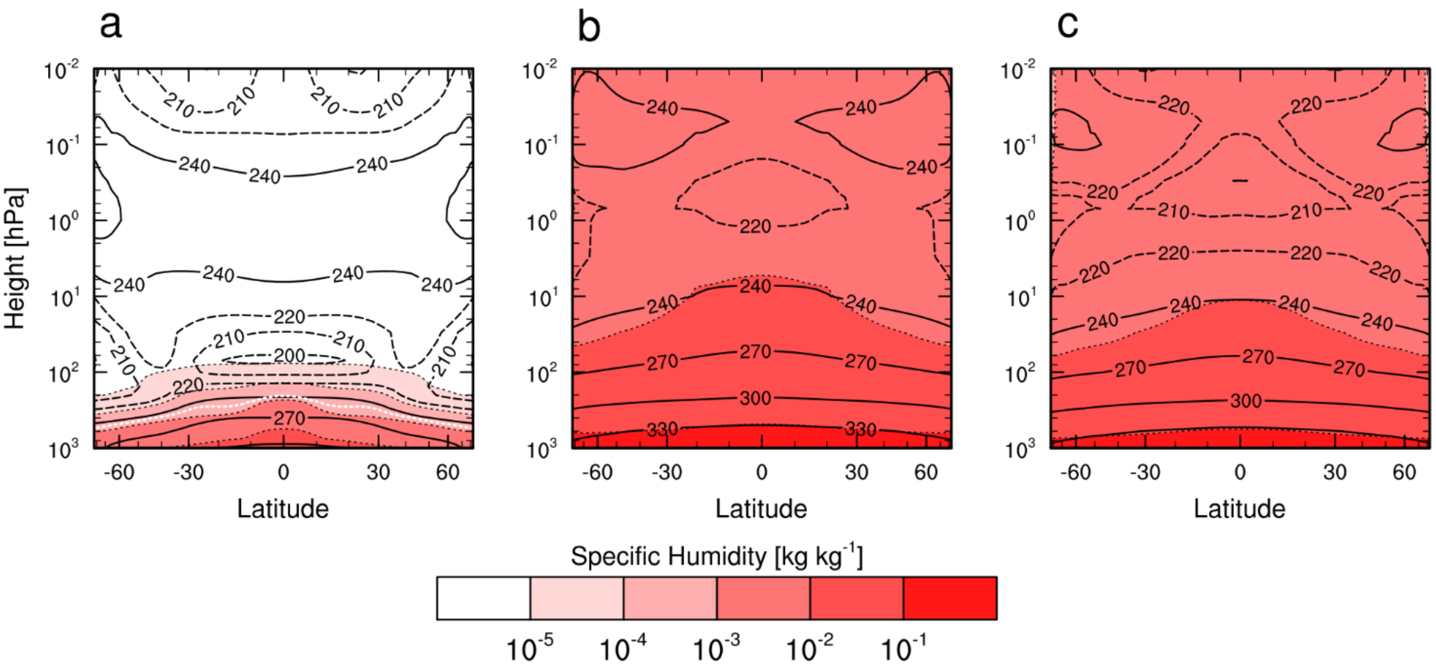

Supplementary Figure 1 | Zonal means of the specific humidity in steady state. Panel a) shows the temporal and zonal means over the last 30 years of simulation of the specific humidity for a TSI of 1.00 S_0, panel b) for a TSI of 1.10 S_0 with the ozone concentrations calculated as described in the methods, and panel c) for the same TSI but with all ozone being removed from the atmosphere. The contours denote temperatures in Kelvin, with the solid lines denoting the contours for 240 K, 270 K, 300 K and 330 K and the dashed lines for the contours of 200 K, 210 K and 220 K. The vertical axes are the height in terms of pressure of dry air and the horizontal axes are the latitudes scaled with their sines.

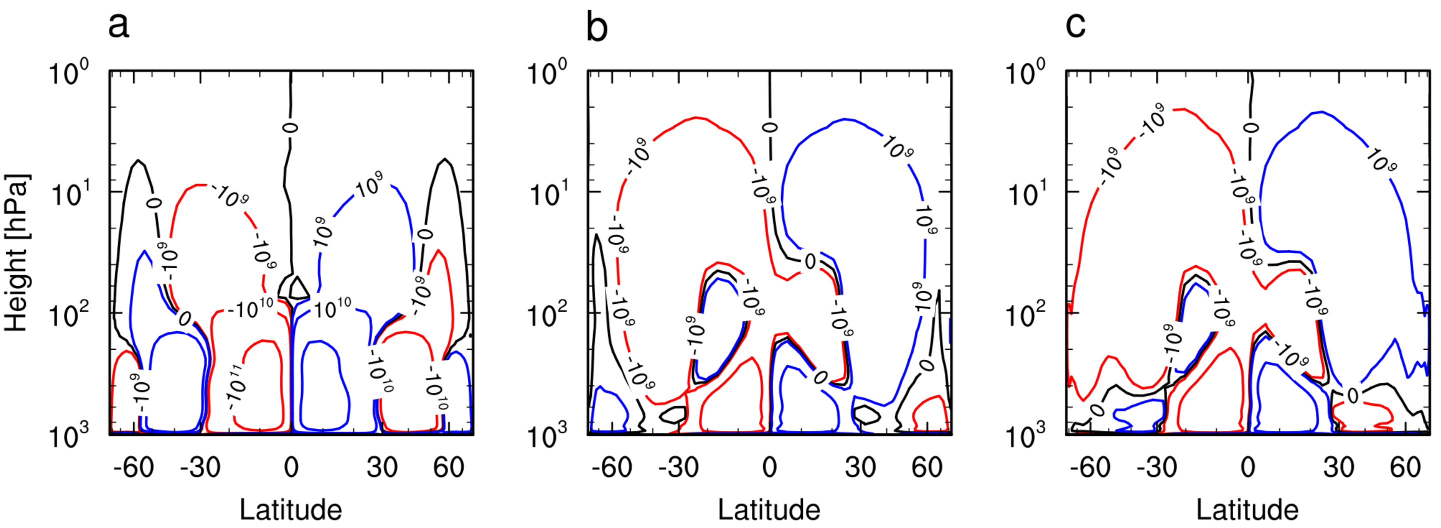

Supplementary Figure 2 | Zonal-mean circulation in steady state. Panel a) shows the temporal mean over the last 30 years of the zonal Eulerian-mean mass-stream function obtained for a TSI of 1.00 S_0, panel b) for a TSI of 1.10 S_0 with the oceanic meridional heat transport prescribed as q-flux, and panel c) for a TSI of 1.10 S_0 with no oceanic meridional heat transport. The vertical axes are the height in terms of pressure of dry air and the horizontal axes are the latitudes scaled with their sines. Blue contour lines denote clockwise rotation and red contour lines denote counter-clockwise rotation.

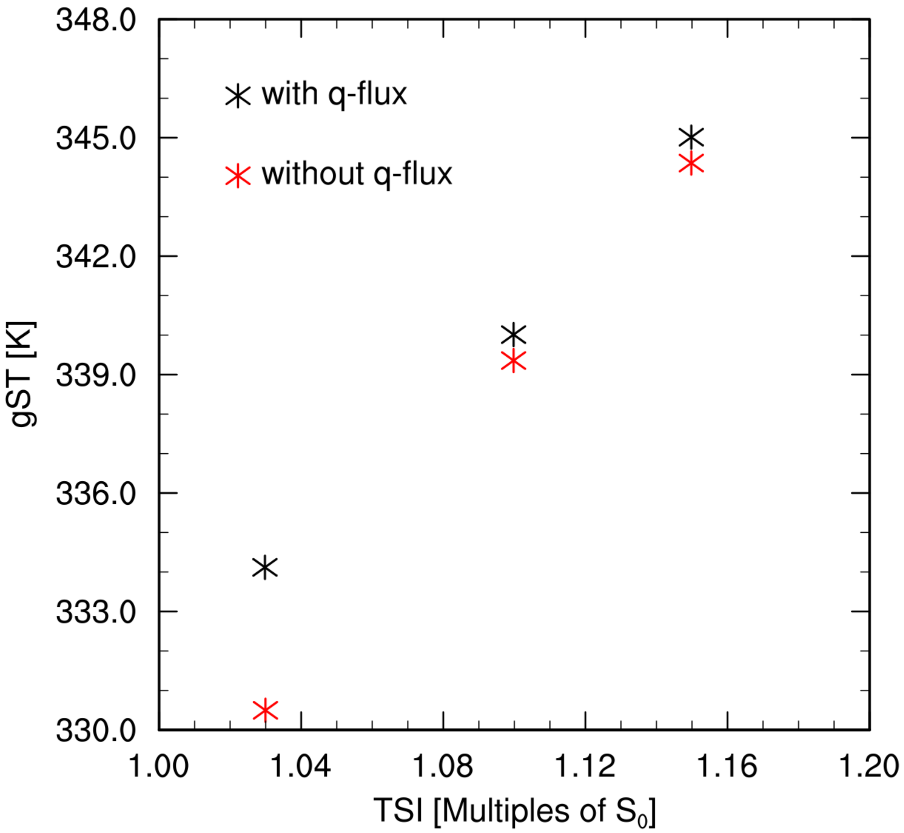

Supplementary Figure 3 | Steady-state gST as a function of TSI for simulations with and without q-flux. The figure shows the temporal average over the last 30 years of gST as a function of TSI for the simulations with solar forcing that attain a steady state in the warm regime. Black marks denote the simulations with q-flux and red marks the ones without.

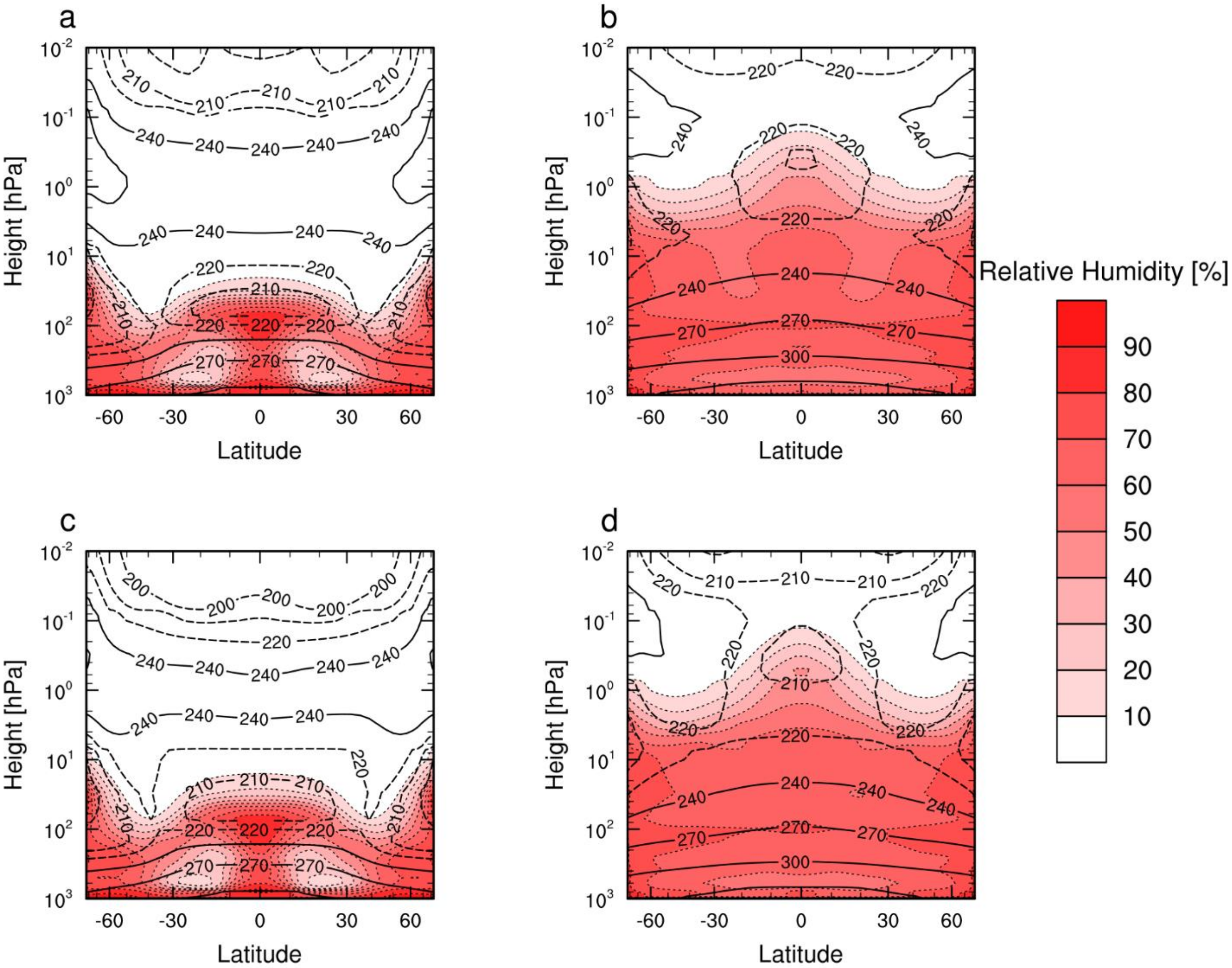

Supplementary Figure 4 | Zonal means of the relative humidity in steady state. Panel a) shows the temporal mean over the last 30 years of the relative humidity for a TSI of 1.03 S_0 in the cold regime and panel b) the mean for the same TSI but in the warm regime. Panel c) shows the same quantity obtained with a TSI of 1.00 S_0 but with atmospheric CO_2 concentrations of 770 ppm in the cold regime and panel d) with the same CO_2 concentration and TSI in the warm regime. The contours denote temperatures in Kelvin, with the solid lines denoting the contours for 240 K, 270 K, 300 K and 330 K and the dashed lines for the contours of 200 K, 210 K and 220 K. The vertical axes are the height in terms of pressure of dry air and the horizontal axes are the latitudes scaled with their sines.
